# A case report: Acute abdominal pain caused by a mesenteric cyst in a 20 years old female patient

**DOI:** 10.1016/j.amsu.2019.12.004

**Published:** 2020-01-03

**Authors:** Irene Cantarero Carmona, José Fernando Trebollé, Daniel Milian García, María José Luesma Bartolomé

**Affiliations:** a Morphological Sciences Department, Anatomy Section, Faculty of Medicine and Nursing, University of Cordoba, 14004, Cordoba, Spain; b General Surgery and Digestive Diseases, Royo Villanova Hospital, 50015, Zaragoza, Spain; c General Surgery and Digestive Diseases, Miguel Servet University Hospital, 50009, Zaragoza, Spain; d Human Anatomy and Histology Department, Faculty of Medicine, University of Zaragoza, 50009, Zaragoza, Spain

**Keywords:** Mesenteric cysts, Laparoscopy technique, Lymph nodes

## Abstract

Mesenteric cysts are documented as rare intra-abdominal benign tumors, whose etiology and classification controversy still exists. They are considered the rarest variety between the abdominal cysts and both its low incidence and the mistaken belief that was a trivial process without apparent symptoms, had contributed to their scarce knowledge. This study aimed to present a mesenteric cyst case with focal acute inflammation and four lymph nodes with follicular lymphoid hyperplasia.

The study was performed 20-year-old female patient, examined in emergency department for abdominal pain 12 hours of evolution located in flank and left upper quadrant, with mild improvement after analgesic treatment, accompanied with mild fever. The patient presented left paramedian cystic formation measuring 4 cm in size with echogenic content inside. Exploratory laparoscopy treatment of emergency was proposed. The postoperative course was favourable remaining afebrile, no abdominal pain and good tolerance to oral intake, so it was hospital discharged within 72 hours of surgery. The diagnosis was mesenteric cyst with focal acute inflammation and four lymph nodes with follicular lymphoid hyperplasia. In conclusion, the type of surgery depended on the size of the cyst, its location in the peritoneal cavity and the experience of the surgeon. Laparoscopy technique was used as a first option.

## Highlights

• Mesenteric cysts are considered the rarest variety within the abdominal cysts.
• The type of surgery depended on the size of the cyst, its location in the peritoneal cavity and the experience of the surgeon.
• Laparoscopy is the technique used as a first option with less comorbidity and postoperative stay than laparotomy.

## 1 Introduction

Mesenteric cysts are rare intra-abdominal benign tumors, whose etiology and classification controversy still exists [1]. They are the rarest variety within the abdominal cysts and both its low incidence and the mistaken belief that it was a trivial process without apparent symptoms, have contributed to their scarce knowledge [2]. The work has been reported in line with the SCARE criteria [3].

## 2 Presentation of case

20 years old woman with no background of interest, who came to the emergency room of the Royo Villanova Hospital due to abdominal pain 12 hours of evolution located in flank and left upper quadrant, with mild improvement after analgesic treatment, accompanied with mild fever. Does not refer nausea or vomiting, or changes in bowel habits.

*Physical exploration*. Temp: 37,6 °C; TA: 123/72; FC: 86 lpm; Sat.O2: 100%. Conscious, oriented, normocolored and normohydrated patient. AC: rhythmic tones to 80 lpm. No audible murmurs or extratones. AP: normoventilated. Abdomen: soft, depressible, painful palpation in the left iliac fossa with no signs of peritoneal irritation. Peristalsis preserved. Bilateral renal succession negative. Femoral pulses present and symmetric. EEII: No edema or signs of DVT. Pedis pulses present.

*Supplementary tests.* CBC: Hb 12,8 g/dl; HCT 37,9%; 216,000 platelets/mm3; Leukocytes 12,100/mm3; Neutrophils 79,4%; Lymphocytes 11,3%. Biochemistry: Glucose 120 mg/dl; Urea 15 mg/dl; Creatinine 0,71 mg/dL; Sodium 140 mEq/L; Potassium 4,3 mEq/L. Coagulation: PT 17,9 seconds; AP 59,3%; INR 1,32; APTT 26,5 seconds; Fibrinogen 358 mg/dl. Urine: No abnormalities. BHCG negative.

*Abdominal ultrasound*. Liver of normal size and morphology with echostructure preserved, without appreciating LOEs. Gallbladder, bile duct, pancreas, spleen and both kidneys in normal size, morphology and echostructure. No lymphadenopathy. No free fluid. Left paramedian cystic formation measuring 4 cm in size with echogenic content inside was identified.

**Fig. 1:**
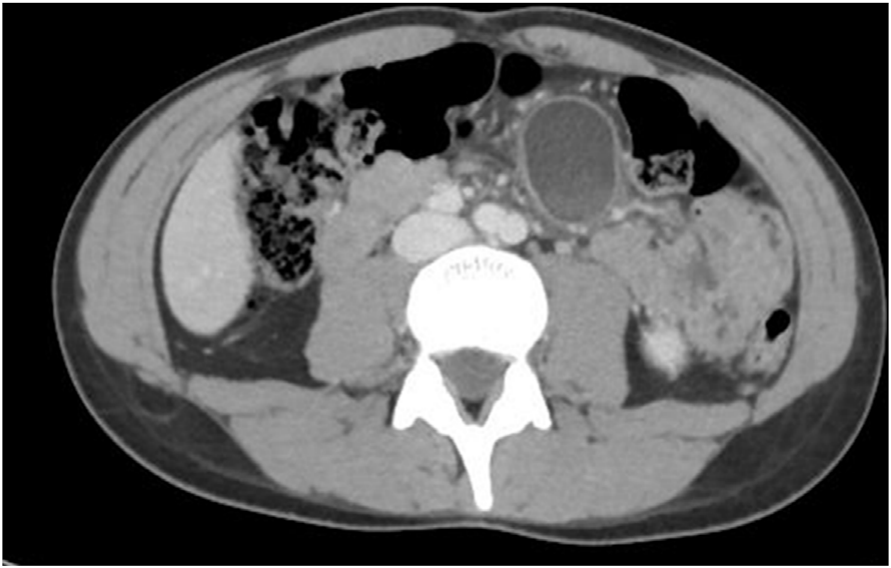
Computerized axial tomography scan which allows observing, Left paramedial cystic injury located at the height of the aortic bifurcation compatible with mesenteric cyst.

*Contrast-enhanced CT*. Left paramedian cystic injury of 4 cm, at the height of the aortic bifurcation with greater density in its bottom portion. It enhances peripherally with the dye and it does not communicate with the gastrointestinal tract, being compatible with complicated mesenteric cyst. The lesion was accompanied by increased density of mesenteric fat (panniculitis) and small locoregional mesenteric lymph nodes. Discrete amount of free fluid in pelvis (Fig. 1).

*Evolution*. Exploratory laparoscopy treatment of emergency was proposed. The postoperative course was favourable remaining afebrile, no abdominal pain and good tolerance to oral intake, so it was hospital discharged within 72 hours of surgery. Anatomopathological examination of the surgical specimen was reported as:

*Macroscopic description.* Formation rounded ovoid 4 × 3 cm maximum dimensions located in the meso of a small intestine loop. After opening it, a cystic cavity of approximately 2 cm of maximum diameter and irregular walls was objective with a whitish colour and soft consistency content.

*Microscopic description*. Cystic lesion was diagnosed on routine histopathological examination and was reported as: Fibroconnective tissue with presence of a cystic cavity without epithelial lining, a proteinaceous and hematic content and with the presence of histiocytic and polymorphonuclear cells. Adjacent to said lesion the presence of four lymph nodes with lymphoid follicular hyperplasia was observed.

**Fig. 2:**
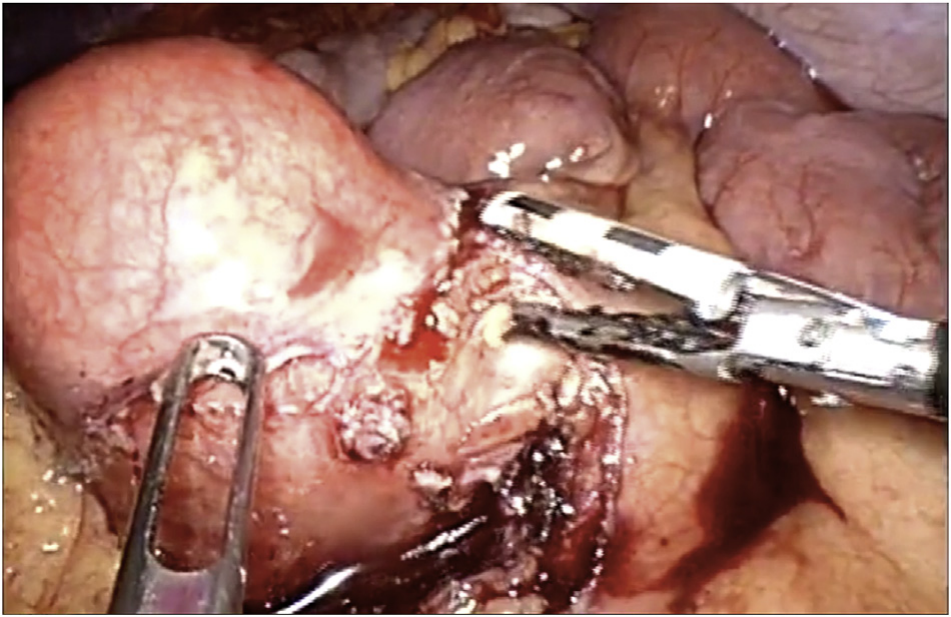
Intraoperative image demonstrating cyst deriving from the mesentery of the small intestine.

*Diagnosis.* Mesenteric cyst was removed before the clinical and radiological findings (Fig. 2). Focal acute inflammation was observed and four lymph nodes with follicular lymphoid hyperplasia. Complete surgical resection removed lymph nodes involvement.

## 3 Discussion

We report the case of a 20-year-old patient who comes to the emergency room with abdominal pain in flank and left upper quadrant associated with mild fever. After performing tests, it was diagnosed “mesenteric cyst with acute focal inflammation and four lymph nodes with lymphoid follicular hyperplasia".

Mesenteric cyst is called to all tumor liquid content of any pathogenetic origin which lies between the two layers of the mesentery, being most frequently on the ileal mesentery and right colon mesentery in 67% and 33% of the cases respectively. Approximately 14,5% corresponds to a retroperitoneal location [4]. Mesenteric cysts, usually were unilocular containing serous fluid. Its size rage from a few millimeters up to completely fill the abdominal cavity. Histologically it includes a cuboid epithelium or cylindrical with microvilli, and sometimes smooth muscle component [5]. The differential diagnosis should include ovarian, pancreatic, renal or splenic cysts, along with hydronephrosis, periapendicular abscesses and even septic ascites, among others [6].

The etiologic of mesenteric cysts is unclear; considered from the injury and obstruction of the lymphatic vessels, degeneration thereof, to the failed fusion of mesenteric layers, being benign proliferation of ectopic lymphatic vessels in the mesentery with the creation of enclosed spaces where the lymph fluid accumulates, the most common cause in most cases [7].

Approximately 50% are asymptomatic. Mild abdominal pain, dull and poorly localized type is the most common clinical manifestation (55–82%). Other manifestations described are nausea and vomiting (45%), abdominal distention (17–61%), abdominal masses palpable (44–61%), constipation (27%) or diarrhea (6%). There have also been cases of compression of adjacent structures, resulting in pyelonephritis, due to ureteral obstruction; acute abdominal because of infection of the tumor; inguinoescrotal tumor; jaundice and anemia due to intratumoral bleeding [8]. Some authors claim that the symptoms associated with this disease are mainly shown in patients whose cysts exceed 5 cm diameter [6,7]. Usually they detected incidentally during ultrasound imaging study, computerized axial tomography or nuclear magnetic resonance, and even during a surgical procedure for another symptom [9].

Regardless of its origin, the treatment of choice is surgical excision, thus avoiding excessive growth or the appearance of serious complications for the patient. Sometimes the cyst closely shares its blood supply with a segment of the intestine so resection intestinal segment involved is required (up to 60% of cases) [9,10].

## 4 Conclusion

The type of surgery depends on the size of the cyst, its location in the peritoneal cavity and the experience of the surgeon. Laparoscopy was the technique used as a first option, since results similar to laparotomy have been achieved but with less comorbidity and postoperative stay. If surgical resection was not possible, another option was the subtotal or partial resection with marsupialization and sclerosis of the endothelium cyst [11].

## Consent of patient

The patient gave written informed consent for publication of this case report.

## Provenance and peer review

Not commissioned, externally peer reviewed.

## Ethical approval

Ethical approval was gained prior to interviews commencing (16/NW/0639).

## Sources of funding

There is no sources of funding.

## Author contribution

Irene Cantarero Carmona: writing the paper.

José Fernando Trebollé: data collection, data analysis or interpretation.

Daniel Milian García: writing the paper.

M^a^ José Luesma Bartolomé: data analysis or interpretation.

## Research registration

Name of the registry:

Unique Identifying number or registration ID:

Hyperlink to the registration (must be publicly accessible):

## Guarantor

Irene Cantarero Carmona: writing the paper.

José Fernando Trebollé: data collection, data analysis or interpretation.

Daniel Milian García: writing the paper.

M^a^ José Luesma Bartolomé: data analysis or interpretation.

## Consent

Written informed consent was obtained from the patient for publication of this case report and accompanying images. A copy of the written consent is available for review by the Editor-in-Chief of this journal on request.

## Declaration of competing interest

There is no conflict of interest.
